# Crosstalk between Integrin αvβ3 and Estrogen Receptor-α Is Involved in Thyroid Hormone-Induced Proliferation in Human Lung Carcinoma Cells

**DOI:** 10.1371/journal.pone.0027547

**Published:** 2011-11-22

**Authors:** Ran Meng, Heng-Yuan Tang, Jennifer Westfall, David London, James H. Cao, Shaker A. Mousa, Mary Luidens, Aleck Hercbergs, Faith B. Davis, Paul J. Davis, Hung-Yun Lin

**Affiliations:** 1 Ordway Signal Transduction, Albany, New York, United States of America; 2 Pharmaceutical Research Institute, Albany College of Pharmacy, Albany, New York, United States of America; 3 Albany Medical College, Albany, New York, United States of America; 4 The Cleveland Clinic, Cleveland, Ohio, United States of America; 5 College of Medicine, King Saud University, Riyadh, Saudi Arabia; Institut de Génomique Fonctionnelle de Lyon, France

## Abstract

A cell surface receptor for thyroid hormone that activates extracellular regulated kinase (ERK) 1/2 has been identified on integrin αvβ3. We have examined the actions of thyroid hormone initiated at the integrin on human NCI-H522 non-small cell lung carcinoma and NCI-H510A small cell lung cancer cells. At a physiologic total hormone concentration (10^−7^ M), T_4_ significantly increased proliferating cell nuclear antigen (PCNA) abundance in these cell lines, as did 3, 5, 3′-triiodo-L-thyronine (T_3_) at a supraphysiologic concentration. Neutralizing antibody to integrin αvβ3 and an integrin-binding Arg-Gly-Asp (RGD) peptide blocked thyroid hormone-induced PCNA expression. Tetraiodothyroacetic acid (tetrac) lacks thyroid hormone function but inhibits binding of T_4_ and T_3_ to the integrin receptor; tetrac eliminated thyroid hormone-induced lung cancer cell proliferation and ERK1/2 activation. In these estrogen receptor-α (ERα)-positive lung cancer cells, thyroid hormone (T_4_>T_3_) caused phosphorylation of ERα; the specific ERα antagonist ICI 182,780 blocked T_4_-induced, but not T_3_-induced ERK1/2 activation, as well as ERα phosphorylation, proliferating-cell nuclear antigen (PCNA) expression and hormone-dependent thymidine uptake by tumor cells. Thus, in ERα-positive human lung cancer cells, the proliferative action of thyroid hormone initiated at the plasma membrane is at least in part mediated by ERα. In summary, thyroid hormone may be one of several endogenous factors capable of supporting proliferation of lung cancer cells. Activity as an inhibitor of lung cancer cell proliferation induced at the integrin receptor makes tetrac a novel anti-proliferative agent.

## Introduction

Thyroid hormone has important roles in regulation of cellular metabolism and of cell proliferation and differentiation [1], [2]. The hormone, usually as 3, 5, 3′-triiodo-L-thyronine (T_3_), stimulates proliferation of a variety of nonmalignant cells, including hepatocytes [3], [4], renal tubular epithelial cells and bone marrow cells [5]. It may inhibit proliferation of certain cells, e.g., fetal cardiomyocytes [6]. We have shown that thyroid hormones induce cell proliferation of several cancer cell lines, including those of breast [7], glioma [8], and the thyroid [9]. Blood vessel cell proliferation is also stimulated by iodothyronines [10]. The thyroid hormone L-thyroxine (T_4_) at physiologic concentrations stimulates angiogenesis and cancer cell proliferation, whereas supraphysiologic levels of T_3_ appear to be required to cause proliferation of such cells [8], [9]. In the case of estrogen receptor (ER)-positive breast cancer cells, we have described dependence of the proliferative effect of thyroid hormone on induction of mitogen-activated protein kinase-dependent serine phosphorylation of ERα that mimics the effect of estrogen [7]. This effect of thyroid hormone can be blocked by the estrogen receptor antagonist, ICI 182,780. Thus, there may be crosstalk between thyroid hormone and estrogen signaling pathways in certain cancer cells; these pathways originate nongenomically outside the nucleus and require ERK1/ERK2, but culminate in specific intranuclear events.

We have recently described a cell surface receptor for thyroid hormone on integrin αvβ3 [11] that is linked to activation of ERK1/ERK2 (extracellular signal regulated kinase 1/2 [ERK1/2]) and, downstream of ERK1/ERK2, to complex transcriptional events, such as tumor cell proliferation [8] and angiogenesis [10]. The integrin is a structural protein of the plasma membrane primarily expressed by rapidly-proliferating cells, namely, cancer cells [12] and dividing blood vessel cells [13], [14]. The protein is essential to the interactions of these cells with extracellular matrix proteins and growth factors [15]. The thyroid hormone receptor is situated near the arginine-glycine-aspartic acid (Arg-Gly-Asp, RGD) recognition site on the integrin [11], [15], [16]. Thus, RGD peptides may interfere selectively with certain thyroid hormone actions initiated at the integrin receptor [17]. At the integrin receptor, tetraiodothyroacetic acid (tetrac) competes with T_4_ and T_3_ to inhibit integrin-initiated actions of the hormones [11], [18]. Derived from T_4_, tetrac is exclusively an inhibitor at the cell surface, but within the cell it has modest thyromimetic activity [19].

In the experiments reported here, thyroid hormone is shown to induce human lung cancer cell proliferation via crosstalk between integrin αvβ3 and ERα. Tetrac consistently blocks this action in two lung cancer cell lines. An estrogen antagonist, ICI 182,780, inhibited integrin αv binding with ERα promoter in the ChIP assay and inhibited ERK1/ERK2 activation and cell proliferation in ERα-bearing lung cancer cells. These results suggest that thyroxine-induced cell proliferation occurs via crosstalk between integrin αvβ3 and ICI 182,780-sensitive signal transduction pathways.

## Materials and Methods

### Cell lines

Human non-small cell lung carcinoma cell line NCI-H522 (ATCC CRL-5810), human small cell lung cancer NCI-H510A (ATCC HTB-184) cells and human ovarian cancer cell line, OVCAR-3, were purchased from ATCC (Rockville, MD). Cells were grown in F12 (NCI-510A) or in RPMI medium (NCI-H522), supplemented with 10% FBS. OVCAR-3 cells were maintained in RPMI1640 supplemented with 20% FBS and insulin. Cells were maintained in a 5% CO_2_/95% O_2_ incubator at 37°C. Prior to treatment, cells were exposed for 2 d to medium that contained 0.25% hormone-stripped fetal bovine serum (FBS).

### Reagents and antibodies

T_4_, T_3_, tetrac and RGD and RGE peptide were obtained from Sigma Chemical Co. (St. Louis, MO). Polyclonal rabbit anti-phosphoERK1/ERK2 (anti-pERK1/pERK2) was purchased from Cell Signaling (Beverly, MA) and monoclonal mouse anti-proliferating cell nuclear antigen (PCNA) was purchased from Santa Cruz (Santa Cruz, CA). Monoclonal mouse anti-ERα, polyclonal rabbit anti-ERβ, monoclonal mouse anti-αvβ3, and monoclonal mouse anti- αvβ5 were purchased from Santa Cruz (Santa Cruz, CA). Goat anti-rabbit IgG and rabbit anti-mouse IgG were obtained from Dako (Carpenteria, CA). The chemiluminescence reagent was from ECL (Amersham, Piscataway, NJ).

### Cell fractionation

Fractionation of cells in a microfuge and preparation of nucleoproteins was by our previously reported methods [20]. Nuclear extracts were prepared by resuspension of the crude nuclei in high salt buffer (hypotonic buffer, 420 mM NaCl and 20% glycerol) at 4°C with rocking for 1 h. The supernatants were collected after subsequent centrifugation at 4°C and 13,000 rpm for 10 min.

### Immunoblotting

The immunoblotting techniques have been standardized in our laboratory [19], [20]. In brief, nucleoproteins were separated on discontinuous SDS-PAGE and then transferred by electroblotting to nitrocellulose membranes (Millipore, Bedford, MA). After blocking with 5% milk in Tris-buffered saline containing 0.1% Tween, the membranes were incubated with various antibodies overnight. Secondary antibodies were either goat anti-rabbit IgG (1∶1000) (Dako, Carpenteria, CA) or rabbit anti-mouse IgG (1∶1000) (Dako), depending upon the origin of the primary antibody. Immunoreactive proteins were detected by chemiluminescence. Blots were quantitated densitometrically.

### RT-PCR

Total RNA was isolated as described previously [9], [10], [11]. First strand complementary DNAs were synthesized from 1 µg of total RNA using oligo dT and AMV Reverse Transcriptase (Promega, Madison, WI). First-strand cDNA templates were amplified for *GAPDH* and *COX-2* mRNAs by polymerase chain reaction (PCR), using a hot start (Ampliwax, Perkin Elmer, Foster City, CA). Primer sequences were *integrin av* (5′- TGGGATTGTGGAAGGAG-3′ and 5′-AAATCCCTGTCCATCAGCAT-3′ [reverse], amplicon size: 329), *integrin β3* (5′-TGCCTCAACAATGAGGTCATCCCT-3′ [forward] and 5′-AGACACATTGAC CACAGAGGCACT-3′ [reverse], amplicon size: 515), *ERα* (5′ ACAAGGGAAGTATGGCTATGGA 3′ [forward] and 5′ GGTCTTTTCGTATCCCACCTTTC 3′ [reverse], amplicon size: 267), ERβ (5′-AAAGCCAAGAGAAACGGTGGGCAT-3′ [forward] and 5′-GCCAATCATGTGCACCAGTTCCT-3′ [reverse], amplicon size: 352) and GAPDH (5′-AAGGTCATCCCTGAGCTGAACG-3′ [forward] and 5′-GGGTGTCGCTGTTGAAGTCAGA-3′ [reverse], amplicon size: 212). The PCR cycle was an initial step of 95°C for 3 min, followed by 94°C for 1 min, 55°C for 1 min, 72°C for 1 min, then 25 cycles and a final cycle of 72°C for 8 min. PCR products were separated by electrophoresis through 2% agarose gels containing 0.2 µg of ethidium bromide/ml. Gels were visualized under UV light and photographed with Polaroid film (Polaroid Co., Cambridge, MA). Photographs were scanned under direct light for quantitation and illustration. Results from PCR products were normalized to the GAPDH signal.

### Chromatin immunoprecipitation and quantitative PCR

ChIP was performed using the EZ ChIP kit (Millipore, MA) according to the manufacturer's instructions. Formaldehyde was directly added to the medium (final concentration 1%) for 10 min to crosslink nuclear proteins with genomic DNA. Then 1.25 M glycine was added for 5 minutes. Cells were washed twice in PBS before being scraped in 1 mL PBS with protease inhibitors (PI) (Complete Mini protease inhibitor cocktail tablets, Roche, IN). Cells were centrifuged for 5 min at 700×g and re-suspended in 1 mL of SDS lysis buffer (1% SDS, 10 mM EDTA, 50 mM Tris, pH 8.1) containing PI. After brief sonication, the resulting supernatant contained DNA fragments ranging from ∼200–1000 bp. Samples were diluted (1∶10) in ChIP dilution buffer (16.7 mM Tris-HCl, pH 8.1; 0.01% SDS; 1.1% Triton X-100, 167 mM NaCl, and 1.2 mM EDTA) containing PI. Samples were pre-cleared with a Protein G Agarose/salmon sperm DNA slurry for 1 h at 4°C. The agarose was pelleted and the supernatant was removed with 10 µl set aside as the “Input”. The supernatant was then allowed to incubate overnight at 4°C with mouse IgG (Dako) or mouse anti-integrin αv (P2W7) (Santa Cruz Biotechnology, CA). 50 µl of Dynabeads Protein G (Invitrogen, CA) were added for 1 h at 4°C with rotation. DNA-protein complexes were recovered from beads by washing with Low Salt Immune Complex Wash Buffer (0.1% SDS, 1% Triton X-100, 2 mM EDTA, 20 mM Tris-HCL, pH 8.1, 150 mM NaCl), High Salt Immune Complex Wash Buffer (0.1% SDS, 1% Triton X-100, 2 mM EDTA, 20 mM Tris-HCL, pH 8.1, 500 mM NaCl), LiCl Immune Complex Wash Buffer (0.25 M LiCl, 1% IGEPAL-CA630, 1% deoxycholic acid, 1 mM EDTA, 10 mM Tris, pH 8.1) (one wash each) and two washes using TE Buffer (10 mM Tris-HCL, 1 mM EDTA, pH 6.0). Samples were eluted twice with 100 µl elution buffer (20 µl 10% SDS, 20 µl 1 M NaHCO_3_ and 160 µl sterile water). To reverse crosslinking, samples were incubated at 65°C for 6 hours or overnight with 8 µl 5 M NaCl. One µl RNase A was added for 30 min at 37°C before incubating at 45°C for 1–2 hours with 4 µl 0.5 M EDTA, 8 µl 1 M Tris-HCL, and 1 µl Proteinase K. DNA was then purified using a Qiagen PCR Purification Kit, and samples then analyzed by Quantitative PCR. 5 µl of DNA were combined with 10 µl of Perfecta SYBR Green FastMix (Quanta Biosciences, MD), 0.3 µl each of 20 µM forward and reverse primers, and 4.7 µl DNase/RNase free water in a MicroAmp™ Optical 384-Well Reaction Plate (Applied Biosystems). The reactions were performed in an ABI Prism 7900 HT SDS instrument (Applied Biosystems) using the following conditions: 2 min at 50°C, 10 min at 95°C, 40 cycles of 15 s at 95°C, and 1 min at 60°C. Data were analyzed with the 7900 HT Sequence Detection Systems Software (version 2.2.3, Applied Biosystems). Primer sequences for promoter of *ERα* were (5′ TAACCTCGGGCTGTGCTCTT 3′ [forward] and 5′ TTCCCTTGGATCTGATGCAGTAG 3′ [reverse]) [21]. Primers for *PIG3* [5′-CAGGACTGTCAGGAGGAGGCGAGTGATAAGG-3′(forward) and 5′-GTGCGATTC-TAGCTCTCACTTCAAGGAGAGG-3′ (reverse)] were used as a negative control for the ChIP assay of integrin αv.

### Thymidine incorporation

Aliquots of cells were incubated with 1 µCi [^3^H]-thymidine (final concentration, 13 nM) in a 24-well culture tray for 16 h. Cells were then washed twice with cold PBS; 5% TCA (1 mL) was then added and the plate was held at 4°C for 30 min. The precipitate was washed twice with cold ethanol, after which 2% SDS (1 ml) was added to each well and the TCA-precipitable radioactivity was quantitated in a liquid scintillation counter.

### Confocal microscopy

Exponentially growing NCI-H522 cells were seeded in a slide chamber. After exposure of cells to medium with 0.25% hormone-stripped FBS for 2 d, cells were treated with 10^−7^ M T_4_, tetrac or both for 24 h. Cells were fixed with 5% formaldehyde in acetone for 5 min, then permeabilized in 100% methanol for 10 min and rehydrated in 90% methanol for 30 min. The cells were incubated with monoclonal antibody to either PCNA or phosphorylated ERα (phospho-ER), followed by Alexa 488-labeled goat anti-mouse antibody and the signal was revealed with the Histostain SP kit, as recommended by the manufacturer (Zymed, South San Francisco, CA). Nuclei were stained with TO-PRO-3 iodide (Molecular Probes, Eugene, OR), and the cells then examined under 250× magnification.

### Data analysis and statistics

Immunoblot and nucleotide densities were measured with a Storm 860 phosphorimager, followed by analysis with ImageQuant software (Molecular Dynamics, Sunnyvale, CA). Student's *t* test, with P<0.05 as the threshold for significance, was used to evaluate the significance of the hormone and inhibitor effects.

## Results

### Thyroid hormone stimulates cell proliferation in human lung cancer cells

**Figure 1:**
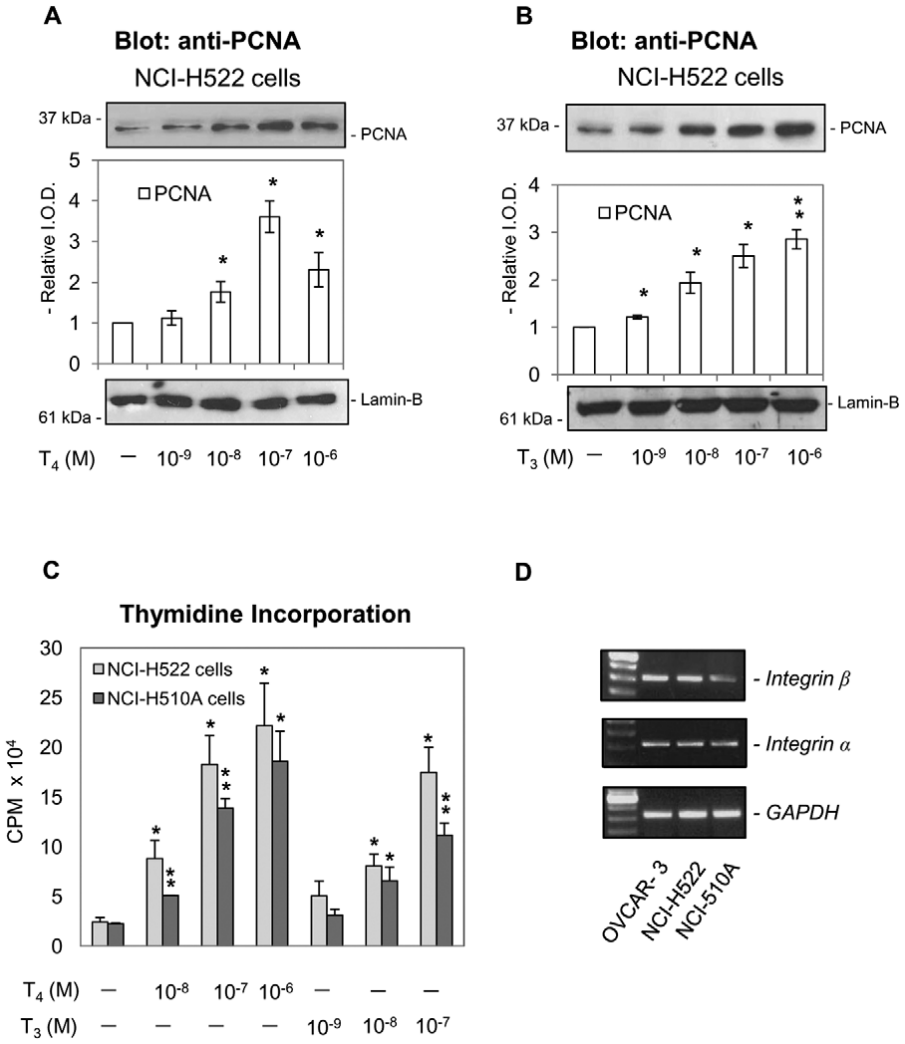
Thyroid hormone stimulates growth of human lung cancer cells in a concentration-dependent manner. **A.** Human non-small cell carcinoma NCI-H522 cells were seeded in 10 mL Petri dishes and cultured in medium containing 0.25% thyroid hormone-depleted and estrogen-depleted serum for 2 d prior to treatment with L-thyroxine (T_4_, 10^−9^–10^−6^ M) for 24 h. T_4_-induced PCNA accumulation was hormone concentration-dependent. Lamin-B immunoblots served as loading controls for the nuclear fractions in these studies and are shown in this figure and the following figures. Reproduced results were conducted from 3 experiments for all experiments. * *p<0.05*, **p<0.01 compared with control. **B.** Cells were similarly cultured with 3, 5, 3′-triiodo-L-thyronine (T_3_, 10^−9^–10^−6^ M) for 24 h. The T_3_ effect on PCNA accumulation was also concentration-dependent. * *p<0.05*, **p<0.01 compared with control. **C.** Human non-small lung cancer NCI-H522 cells and human small cell lung cancer NCI-H510A cells were treated with T_4_ (10^−8^ to 10^−6^ M) or T_3_ (10^−9^ to 10^−7^ M). [^3^H]-Thymidine was added with hormone for 24 h prior to harvest for assay of thymidine incorporation. In these studies, both hormones stimulated thymidine incorporation. At physiologic total hormone concentrations, however, T_4_ (10^−7^ M) was more effective than T_3_ (≤10^−9^ M). (* *p<0.05*, **p<0.01 compared with untreated controls.) **D.** Total RNA was extracted from OVCAR-3, NCI-H522 and NCI-H510A cells and prepared for RT-PCR as described in Materials and Methods. OVCAR-3 cells served as positive controls. Both NCI-H522 and NCI-H510A cells expressed integrin αvβ3, although NCI-H510A cells expressed less β3 than NCI-H522 cells.

In a concentration-dependent manner, T_4_ induced PCNA accumulation in NCI-H522 non-small cell lung carcinoma cells (Fig. 1A). Maximum effects were seen at 10^−8^ to 10^−7^ M total hormone concentration; in the medium/buffer systems we use, such concentrations yield physiological free T_4_ levels [11]. T_3_ also increased PCNA abundance in NCI-H522 cells (Fig. 1B), but the effective hormone concentrations exceeded physiologic levels. Confirmation of the effects of T_4_ and T_3_ on PCNA was obtained in thymidine incorporation studies in non-small cell NCI-H522 lung cancer cells as well as in small cell NCI-H510A cancer cells (Fig. 1C). Because we have shown that thyroid hormones bind to a receptor on integrin αvβ3, we investigated whether integrin αvβ3 was expressed in NCI-H522 and NCI-H510A cells. Human ovarian OVCAR-3 cells were used as a positive control [22]. Results shown in Fig. 1D indicate that both lung cancer cell lines express integrin αvβ3. Although integrin αv is expressed to a similar degree in both cell lines, the expression of integrin β3 in NCI-H510A cells is far less than that in NCI-H522 cells.

### Integrin αvβ3 contributes to thyroid hormone-induced proliferation in lung cancer cells

**Figure 2:**
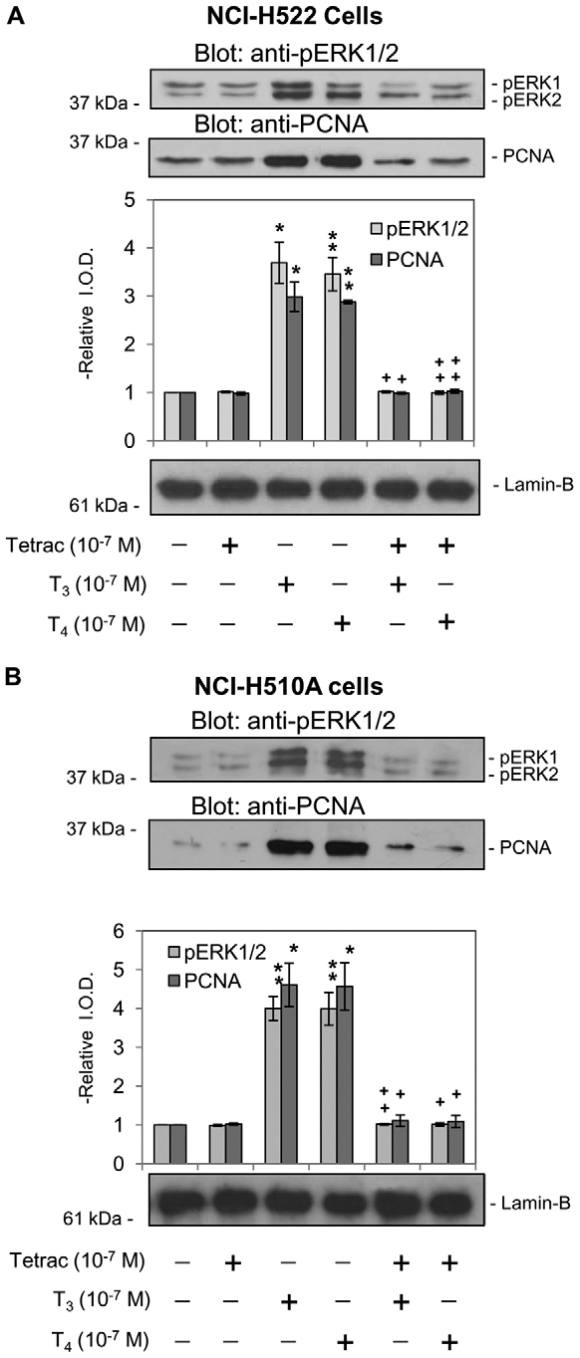
Tetrac inhibits thyroid hormone-induced PCNA accumulation and ERK1/2 activation in different lung carcinoma cells. **A.** non-small cell NCI-H522 and **B.** small cell NCI-H510A lung carcinoma cells were treated with tetrac (10^−7^ M) for 30 min prior to addition of T_3_ (10^−7^ M) or T_4_ (10^−7^ M). Cells were harvested either 30 min (ERK1/2) or 24 h (PCNA) after the onset of treatment. Tetrac, alone, did not induce either ERK1/2 activation or PCNA expression; however, tetrac did block the effects of both T_4_ and T_3_ in these two cell lines by inhibition of thyroid hormone binding to the cell surface integrin receptor. * *p<0.05*, **p<0.01 compared with control; **+** *p<0.05*, ++*p<0.01* comparing hormone effects with and without tetrac.

Non-small cell lung carcinoma NCI-H522 cells were pretreated with 10^−7^ M T_4_ or T_3_, in either the presence or absence of tetrac. Thyroid hormone-induced PCNA expression and ERK1/2 activation were blocked by tetrac (Fig. 2A). Tetrac itself did not induce PCNA expression. Similar results were observed in small cell lung cancer NCI-H510A cells in which tetrac blocked thyroid hormone-induced ERK1/2 activation and PCNA expression (Fig. 2B).

**Figure 3:**
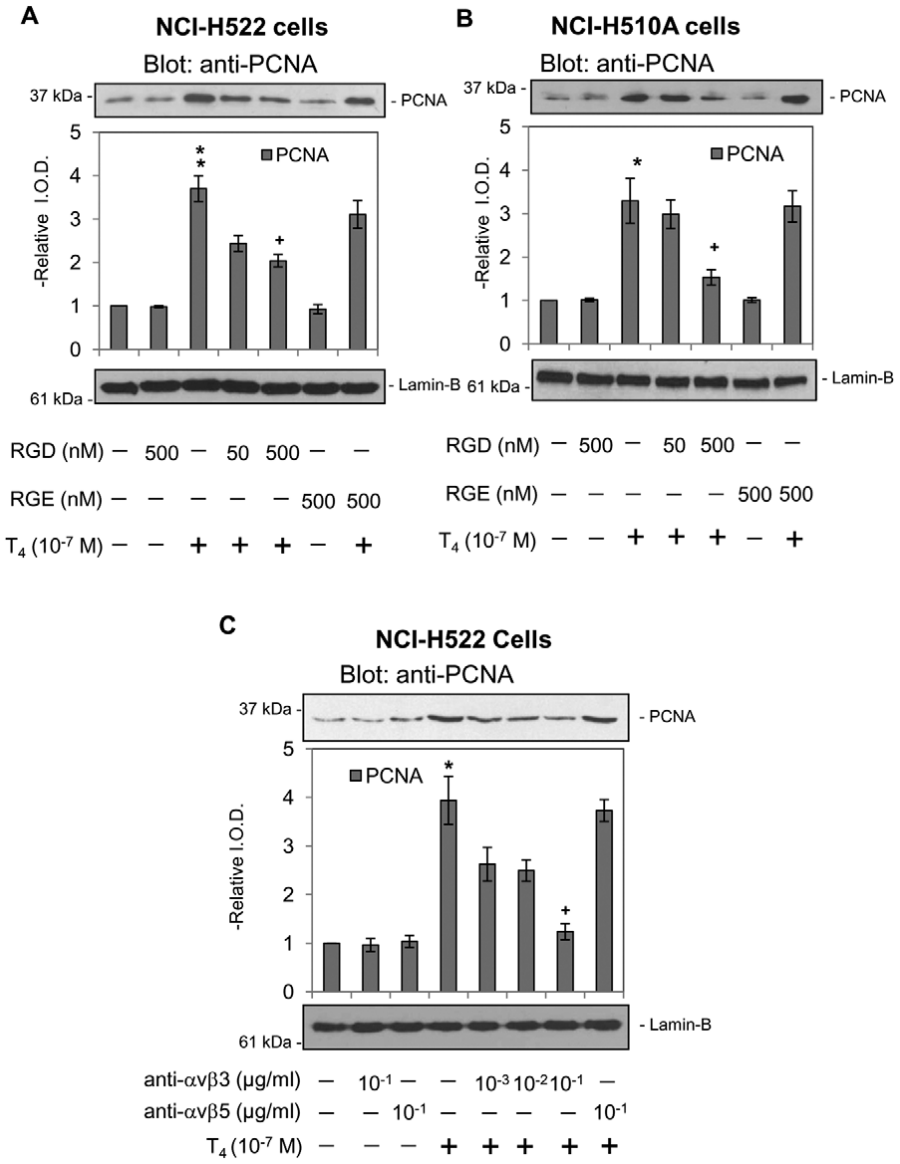
Integrin αvβ3 mediates T_4_-induced PCNA accumulation in lung cancer cells. **A.** Non-small cell carcinoma NCI-H522 cells and **B.** small cell NCI-H510A cells were each treated with T_4_ (10^−7^ M) in the presence or absence of RGD peptide (50–500 nM) or RGE peptide (500 nM) for 24 h. T_4_-induced PCNA expression was inhibited by the RGD peptide, but not by a control (RGE) peptide, indicating that interaction with the integrin at or near the RGD binding site is required for the proliferative effect of thyroid hormone to be seen. * *p<0.05*, **p<0.01 compared with control; **+** *p<0.05* compared with cells treated without RGD. **C.** Non-small cell carcinoma NCI-H522 cells were each treated with anti-αvβ3 (0.001 µg/ml–0.1 µg/ml) or anti-αvβ5 (0.1 µg/ml) antibody for 24 h and then with T_4_ (10^−7^ M) for 24 h. Anti-αvβ3, but not anti-αvβ5, blocked T_4_-induced PCNA accumulation. This effect was antibody dose-responsive. Lamin-B immunoblots served as loading controls for the nuclear fractions used in these assays. * *p<0.05*, **p<0.01 compared with control; **+** *p<0.05* compared with and without anti-αvβ3.

NCI-H522 cells were pretreated with either RGD peptide or RGE peptide for 24 h prior to incubation with 10^−7^ M T_4_ for 24 h. The RGD peptide inhibited thyroid hormone-induced PCNA expression by these non-small cells in a dose-responsive manner, whereas the control RGE peptide had no effect on T_4_-induced PCNA expression (Fig. 3A). Similar results were observed in small cell lung cancer NCI-H510A cells (Fig. 3B). We further confirmed that the integrin αvβ3 *is* the plasma membrane binding site for T_4_ by treatment of cells with anti-integrin αvβ3. Integrin αvβ5 antibody served as a control. Only the integrin αvβ3 antibody blocked thyroid hormone-induced PCNA expression in NCI-H522 cells (Fig. 3C). These results and other recent studies of the integrin [16], [17], [18] implicate the plasma membrane integrin thyroid hormone receptor in the proliferative effects of iodothyronines on cancer cells and on angiogenesis.

### Thyroid hormone causes phosphorylation of ERα and transfer of that receptor from the cytosol to nuclei of treated cells

**Figure 4:**
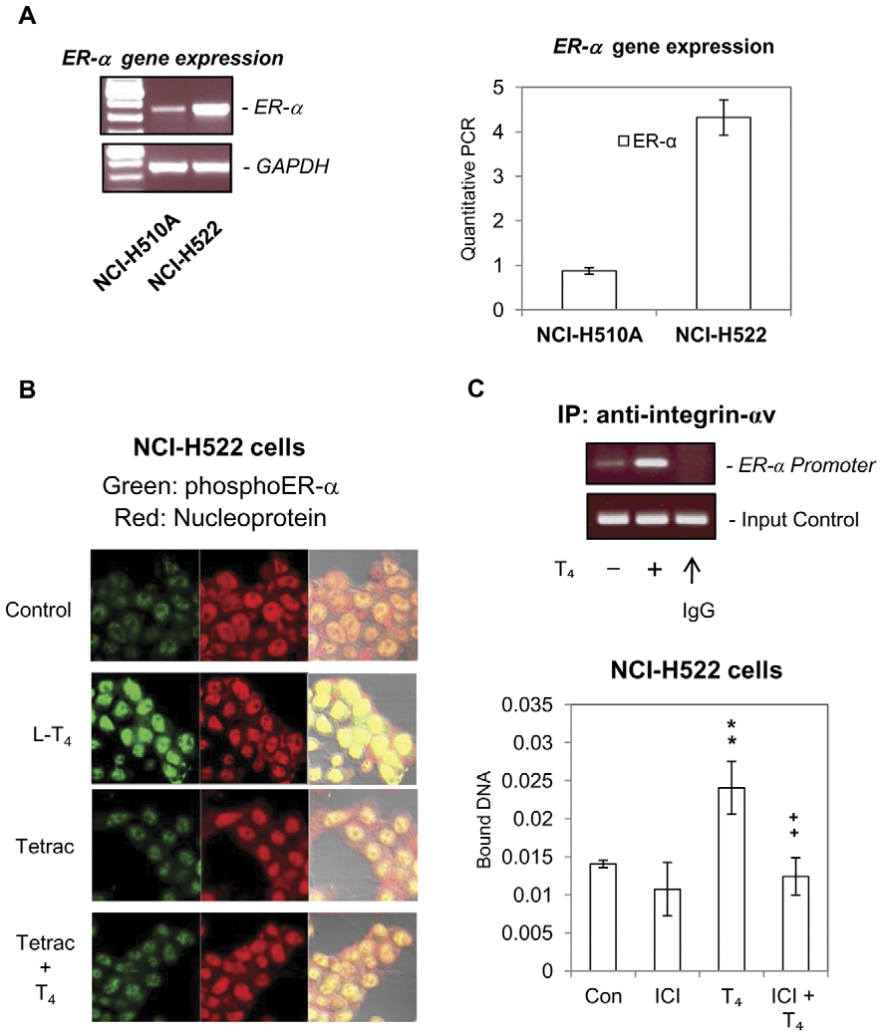
T_4_ stimulates nuclear accumulation of phosphorylated ERα. **A.** The expression of ERα in NCI-H522 non-small cell lung cancer cells and small cell NCI-H510A cells, studied by RT-PCR, revealed that the NCI-H522 cells contained more ERα than the NCI-H510A cells. **B.** Non-small cell NCI-H522 cells were treated with T_4_ (10^−7^ M) and/or tetrac (10^−7^ M) for 24 h, and then examined by confocal microscopy. Nuclear chromosomal material is indicated by the red color and phosphorylated ERα (pERα) by a green color. T_4_ caused accumulation of phosphoERα (pERα, green) in cell nuclei (red) producing a yellow color due to the superimposition of red and green. With the addition of tetrac, the nuclear accumulation of pERα was less evident in T_4_-treated cells, while tetrac, alone, did not cause an increase in nuclear accumulation of ERα. Cells were viewed at 250× magnification. **C.** Cells were treated with or without T_4_ (10^−7^ M) for 24 h, then fixed and harvested for ChIP as described in **Materials and Methods**. Immunoprecipitation was performed with anti-integrin αv antibody and mouse IgG was used as a negative control (**upper panel**). Cells were treated with T_4_ (10^−7^ M) in the presence or absence of ICI (5 nM) for 24 h, then fixed and harvested for ChIP as described in **Materials and Methods**. Immunoprecipitation was performed with anti-integrin αv antibody. The primer used was the ERα promoter sequence. **p<0.01 compared with control; ++*p<0.01* comparing samples with and without ICI.

The estrogen receptor ERα is variably expressed in non-small cell and small cell lung cancer cell lines, as shown in Fig. 4A. NCI-H522 cells expressed a greater level of this receptor with 2–5 times the level in the NCI-H510A non-small cell line (Fig. 4A). Thyroxine is known to induce ERα phosphorylation in human breast cancer MCF-7 cells [7]. We demonstrate by confocal microscopy in Fig. 4B that thyroid hormone causes nuclear accumulation of phosphorylated ERα in non-small cell NCI-H522 lung cancer cells. The nuclear accumulation of phosphorylated ERα caused by T_4_ (shown in green) co-localized with nuclear chromatin (shown in red), yielding a yellow color. This action of T_4_ was inhibited by co-incubation of cells with T_4_ and tetrac, although tetrac alone had little effect (Fig. 4B). A specific inhibitor of ERα activation, ICI 182,780 (ICI), blocks thyroid hormone-stimulated thymidine uptake in human breast cancer MCF-7 cells, suggesting that T_4_-induced proliferation of MCF-7 cells is dependent on the presence of ERα [7]. Thyroid hormone (T_4_) induces integrin αv translocation into nuclei and association with p300 [23] suggesting that nuclear integrin αv may play a role in thyroid hormone-dependent gene transcription. In order to examine the relationship between integrin αv and ERα on gene regulation, NCI-H522 cells were treated with T_4_ in the presence or absence of ICI. A chromatin immunoprecipitation assay (ChIP) was conducted by using anti-integrin αv antibody. Mouse IgG was used as a negative control. T_4_ increased integrin αv binding to the *ERα* promoter *except* in the sample immunoprecipitated with mouse IgG (Fig. 4C, upper panel). The binding of integrin αv to the *ERα* promoter sequence is specific, as with the use of primer sequences of the *PIG3* promoter we were unable to produce a visible DNA sequence (results not shown). The T_4_-activated integrin αv formed a complex with the *ERα* promoter, which was reduced by ICI (Fig. 4C, lower panel). These results suggest that crosstalk between integrin αvβ3 and ERα is involved in T_4_-dependent transcription.

### Phosphorylation of nuclear ERα parallels thyroid hormone-induced proliferation in lung cancer cells

**Figure 5:**
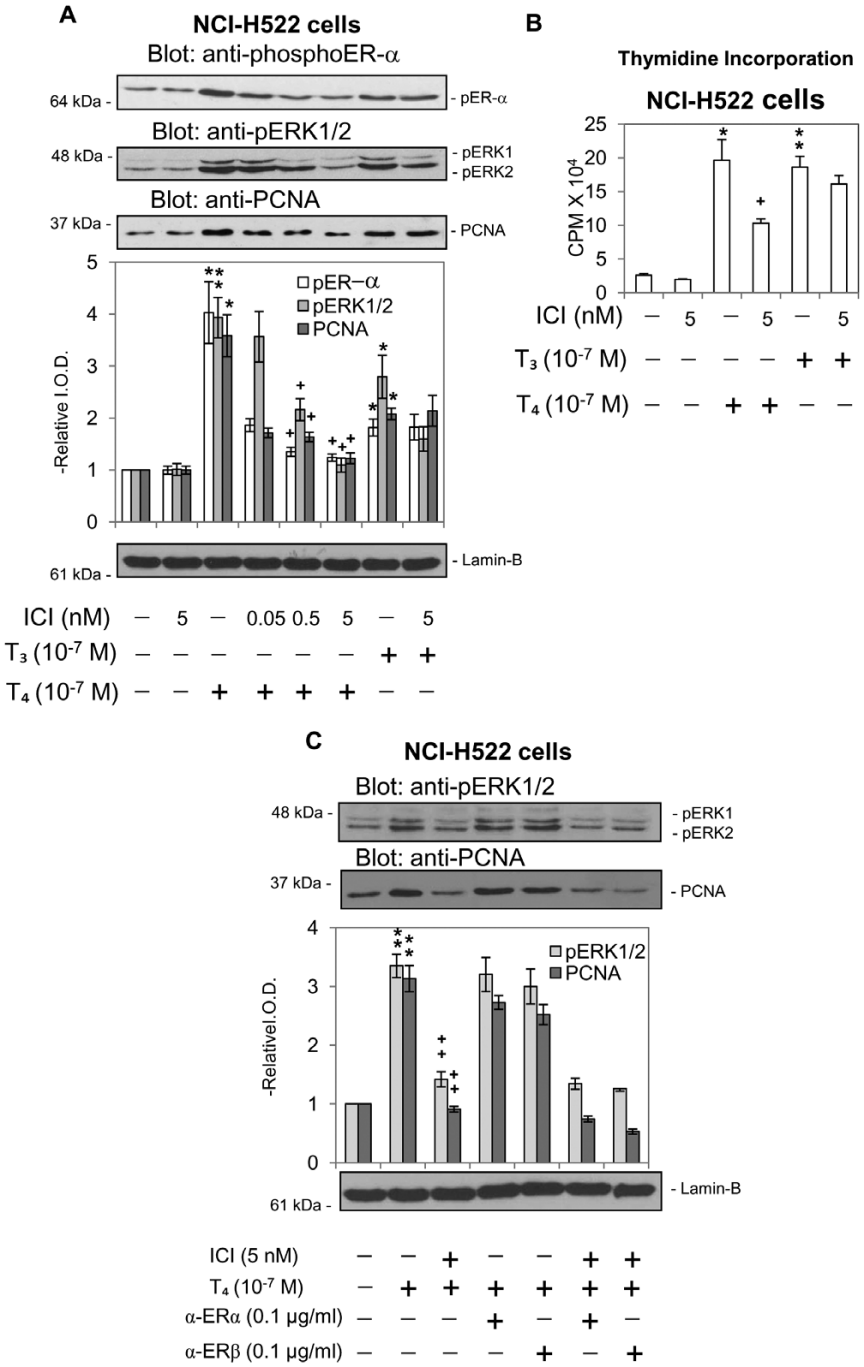
Effect of ICI 182,780 on cell proliferation induced by T_4_ in NCI-H522 cells. **A.** Non-small cell lung cancer NCI-H522 cells were treated with T_3_ or T_4_ (10^−7^ M, 24 h) in the presence or absence of ICI 182,780 (ICI), an ER antagonist (0.05–5 nM). ICI inhibited phosphorylation of ERα, ERK1/2 activation, and the proliferative effect (PCNA) of T_4_ in these cells. However, the action of T_3_ was minimally affected by ICI, except for diminution in pERK1/2 levels. * *p<0.05*, **p<0.01 compared with control; **+** *p<0.05* comparison with and without ICI. **B.** NCI-H522 cells cultured in 0.25% hormone-stripped serum-containing medium for 2 d were then placed in 10% stripped serum-containing medium and treated with ICI (5 nM) for 30 min prior to treatment with thyroid hormone (10^−7^ M T_4_ and T_3_) for 24 h. 1 µCi [^3^H]-thymidine was added simultaneously with thyroid hormone for 24 h prior to harvest for radiolabeled thymidine incorporation assay. The results of these studies confirm the studies in Fig. 5A, showing that T_4_-induced cell proliferation is dependent on activation of ERα, whereas cell proliferation stimulated by T_3_ is independent of ERα phosphorylation. * *p<0.05*, **p<0.01 compared with control; ++*p<0.01* compared with and without ICI. **C.** NCI-H522 cells were treated with anti-ERα or -ERβ antibody (0.1 µg/ml) for 24 h and ICI (5 nM) for 30 min prior to treatment with T_4_ (10^−7^ M) for either 30 min or 24 h. Nuclear proteins were separated by SDS-PAGE and western blotting analyses carried out with anti-pERK1/2 or -PCNA antibodies. Thyroid hormone-induced ERK1/2 activation and PCNA accumulation was affected by ICI but not by either anti-ERα or anti-ERβ antibody. **p<0.01 compared with control; ++*p<0.01* compared with and without ICI.

We further studied the role of ERα on thyroid hormone-induced proliferation in lung cancer cells. As shown in Fig. 4A both NCI-H522 and NCI-H510A lung cancer cell lines express ERα (NCI-H522≫NCI-H510A). When non-small cell carcinoma NCI-H522 cells were treated with thyroid hormone (T_4_ or T_3_) for 24 h in the presence or absence of 0.05–5 nM ICI, both hormones caused activation of ERK1/2 and PCNA accumulation (Fig. 5A). The T_4_ effects on nuclear accumulation of pERα, pERK1/2 and PCNA were all sensitive to increasing concentrations of ICI, indicating a direct association with activation (phosphorylation) of ERα. In contrast, T_3_ minimally increased ERα phosphorylation and cell proliferation, and these were not affected by ICI; this inhibitor only suppressed ERK1/2 activation by T_3_. Similar results were observed by using another inhibitor of ERα, tamoxifen, which inhibited T_4_-induced ERK1/2 activation and PCNA accumulation (results not shown). Findings in the thymidine incorporation studies (Fig. 5B) paralleled results of PCNA immunoblots in that cell proliferation was inhibited by ICI in the presence of T_4_, but not in the presence of T_3_.

Studies have shown that ERα also exists on the cell membrane [24], [25]. To exclude the possibility that membrane ERα is activated by thyroid hormone and translocates into the nucleus, experiments were conducted to examine if membrane ERα is involved in thyroxine's action. NCI-H522 cells were pre-treated for 24 h with anti-ERα or anti-ERβ as controls prior to addition of ICI and 10^−7^ M T_4_. Cells were harvested and activation of ERK1/2 and increased expression of PCNA by T_4_ was evaluated. T_4_-induced ERK1/2 activation and PCNA expression was inhibited by ICI but not by either anti-ERα or anti-ERβ antibody (Fig. 5C). The combination of ICI and antibody did not alter the inhibitory effect of ICI. These results indicated that the T_4_-induced signal activates cytosolic ERα and translocates phosphorylated ERα into nuclei accompanied by activated ERK1/2.

**Figure 6:**
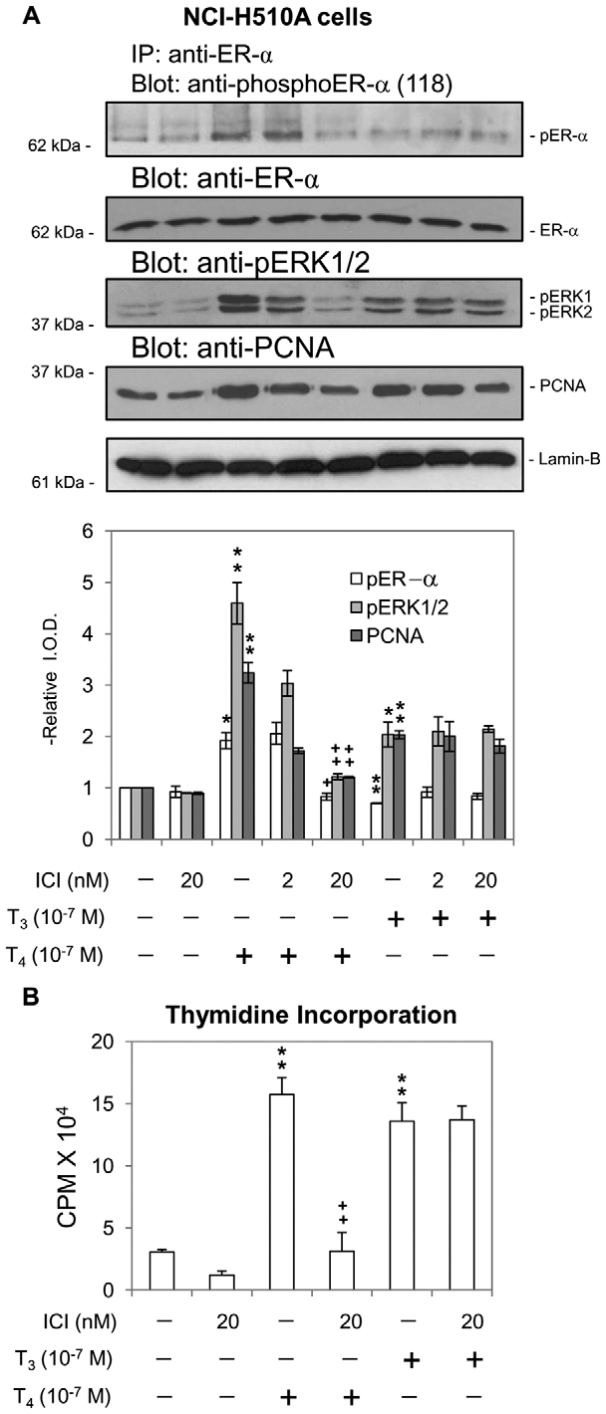
Effect of ICI 182,780 (ICI) on cell proliferation induced by T_4_ in NCI-H510A cells. **A.** Small cell carcinoma NCI-H510A cells, which express low levels of ERα, were treated with T_4_ or T_3_ (10^−7^ M, 24 h) in the presence or absence of ICI (2 or 20 nM). This inhibitor suppressed ERK1/2 activation, phosphorylation of ERα and cell proliferation by T_4_—but not by T_3_—in NCI-H510A cells. ICI did not inhibit any actions of T_3_. * *p<0.05*, **p<0.01 compared with control. **+** *p<0.05*, ++*p<0.01* compared with and without ICI. **B.** NCI-H510A cells were treated with ICI (20 nM) for 30 min prior to treatment with T_4_ or T_3_ (10−7 M) for 24 h in the presence of radiolabeled thymidine. The results of these studies in small cell lung cancer cells are similar to the results of studies in non-small cell lung cancer cells (Fig. 5), again showing that the proliferative action of T_3_, in contrast to that of T_4_, is not mediated by activation of ERα, in contrast to the proliferative action of T_4_ which is blocked by ICI, the ERα inhibitor. **p<0.01 compared with control; ++*p<0.01* compared with and without ICI.

Effects of the ICI compound were also studied in small cell carcinoma (NCI-H510A) cells (Fig. 6A). Because these cells contain less ERα than the NCI-H522 cells, immunoprecipitation of ERα and subsequent protein electrophoresis were used to enhance the content of ERα present in experimental samples. Both T_4_ and T_3_ induced ERK1/2 activation and PCNA expression, but only T_4_ caused ERα phosphorylation in these cells (Fig. 6A). The T_4_-induced effects were all inhibited by ICI, whereas there was little effect of ICI on T_3_-induced ERK1/2 activation and PCNA accumulation. Similar results were observed with thyroid hormone-induced thymidine incorporation studies (Fig. 6B) in which the proliferative effect of T_4_ was inhibited by ICI, but cell proliferation in the presence of T_3_ was not affected by ICI. The differences between the effects of T_4_ and T_3_ in NCI-H510A cells were not surprising, given our recent description of the complexity of the thyroid hormone binding site on the integrin [16] and the ability of the receptor to distinguish between T_4_ and T_3_ and to generate discrete downstream signals in response to the two thyroid hormones. These results in NCI-H510A cells do indicate that T_3_ stimulates cell proliferation by a mechanism that is independent of ERα.

## Discussion

Acting at the cell surface integrin αvβ3 receptor for thyroid hormone, T_4_ and T_3_ induced cell proliferation in the human lung cancer cell lines studied in the present report. Thyroid hormone-induced proliferation in these cells is blocked by tetrac, an analogue of thyroid hormone which has been shown to interfere with the binding of the thyroid hormones T_4_ and T_3_ to integrin αvβ3 [11]. This hormonal action on lung cancer cells is also blocked by RGD peptide and by antibody to integrin αvβ3, confirming that cell surface integrin αvβ3 is the initiation site of the proliferative action of thyroid hormone. These lung cancer results are consistent with our previous observations of induction by thyroid hormones of cell proliferation in human breast cancer [7], human thyroid cancer [9], human glioma [26] and rat glioma cells [8]. In such tumor cells, physiologic concentrations of endogenous thyroid hormone, particularly T_4_, may serve as a growth factor.

There is crosstalk between integrin αvβ3 and several polypeptide growth factor receptors on the plasma membrane that appear to be clustered with the integrin [27], [28], [29]. Thyroid hormone also nongenomically affects the behavior of the epidermal growth factor receptor [30]. Furthermore, we have previously shown that tetrac modulates such crosstalk between hormone and growth factor in the case of TGFα as well [30]. In addition to crosstalk, however, thyroid hormone can nongenomically signal downstream within the cell to the classic nuclear thyroid hormone receptor (TR); that is, from the cell surface, the hormone can alter the activity state of a nuclear thyroid hormone receptor (TRβ1) via control of ERK1/ERK2-mediated serine phosphorylation of the nucleoprotein [25], [31].

What was somewhat surprising in studies of downstream signaling induced by T_4_ several years ago was that this hormone could, through ERK1/2, trespass into the domain of steroids and promote phosphorylation of Ser-118 of ERα in human breast cancer cells [7]. This mimicked precisely the action of estradiol and was important to the proliferative action of thyroid hormone on these breast cancer cells. A natural extension of this finding was to examine the possibility that in ERα-bearing lung cancer cells, the proliferative effect of thyroid hormone was also ER-mediated. The present studies confirm that this is the case for T_4_. On the other hand, while T_3_ can stimulate lung cancer cell proliferation at above-physiologic hormone concentrations, this action of T_3_ appears *not* to require ER. We have recently shown that the integrin receptor for thyroid hormone readily distinguishes between T_4_ and T_3_ and can generate different or similar signals from each hormone that result in discrete or similar downstream biologic effects [17], [18]. We have also recently demonstrated that the androgen dihydrotestosterone can stimulate breast cancer cell proliferation by an ERα-dependent mechanism when the receptor is present and an ERα-independent process when tumor cells lack that receptor [32].

It is clear that TR in the nuclear compartment does not play a primary role in the integrin αvβ3-initiated actions of thyroid hormone [1]. However, overexpression of TRβ1 can be involved in thyroid hormone (T_3_)-induced *inhibition* of proliferation of certain cells [33] and mutations of nuclear TR can result in interesting models of thyroid carcinoma [1]. In addition, nuclear TR isoforms that reside in cytoplasm can participate in nongenomic actions of thyroid hormone [34], [35], at least one of which modulates downstream the expression of the *hypoxia-inducible factor- 1α* (*HIF-1α*) gene [36]. We have also shown that thyroid hormone can act at the cell surface on the integrin receptor and influence expression of *HIF-1α* [17].

Further understanding of the integrin receptor for thyroid hormone may permit the receptor to emerge as an anti-proliferation target. Because of the anti-angiogenic properties of tetrac that are expressed via the αvβ3 receptor, this hormone analogue has more than one attractive feature in the setting of cancer [17]. The agent may also chemosensitize certain tumor cells that are chemoresistant [37]. The present studies suggest that certain lung cancers may be susceptible to therapeutic agents that act at the integrin iodothyronine receptor, such as tetrac.
